# Brain magnetic resonance imaging in the DE50-MD dog model of Duchenne muscular dystrophy reveals regional reductions in cerebral gray matter

**DOI:** 10.1186/s12868-023-00788-2

**Published:** 2023-03-17

**Authors:** Abbe H. Crawford, Natasha L. Hornby, Alerie G. de la Fuente, Richard J. Piercy

**Affiliations:** 1 Comparative Neuromuscular Diseases Laboratory, Department of Clinical Science and Services, Royal Veterinary College, London, UK; 2 Institute of Health and Biomedical Research of Alicante (ISABIAL), Alicante, Spain; 3 Institute of Neurosciences CSIC-UMH, San Juan de Alicante, Spain; 4 Wellcome-Wolfson Institute for Experimental Medicine, Queen’s University, Belfast, UK

**Keywords:** Duchenne muscular dystrophy, Canine, Brain, Magnetic resonance imaging

## Abstract

**Background:**

Duchenne muscular dystrophy is a X-linked disease characterized by severe and progressive muscle weakness, alongside cognitive impairment and a range of neurobehavioral disorders secondary to brain dystrophin deficiency. Duchenne muscular dystrophy patients have reduced cerebral gray matter and altered white matter ultrastructure (detected by magnetic resonance imaging) compared to age-matched controls.

**Methods:**

We studied the DE50-MD canine model of Duchenne muscular dystrophy, which is deficient in full length brain dystrophin (Dp427) isoforms and has a neurocognitive phenotype. Eight DE50-MD and 6 age-matched littermate wild type male dogs underwent serial brain magnetic resonance imaging from 14 to 33 months of age.

**Results:**

Reduced regional gray matter was detected in DE50-MD dogs compared with wildtype, including the piriform lobe, hippocampus and cingulate gyrus. Lateral ventricle volume was larger in DE50-MD dogs. Differences did not progress over time. White matter volume did not differ between DE50-MD and wildtype dogs. There was no difference in brain nor cranial vault volume between DE50-MD and wildtype dogs.

**Conclusion:**

Dystrophin deficiency in the canine brain results in structural changes that likely contribute to the neurocognitive phenotype.

**Supplementary Information:**

The online version contains supplementary material available at 10.1186/s12868-023-00788-2.

## Background

Duchenne muscular dystrophy (DMD) is an X-linked disease characterized by progressive muscle weakness due to mutations in the *DMD* gene. The *DMD* gene encodes dystrophin, a protein expressed in striated muscle where it provides structural stability by linking the actin cytoskeleton to the extracellular matrix. In addition to muscle weakness, DMD is associated with cognitive impairment and a range of neurobehavioral disorders secondary to dystrophin deficiency in the brain. Approximately 30% of boys with DMD have intellectual impairment (IQ < 70) [1], alongside a higher incidence of attention-deficit/hyperactivity disorders, anxiety disorders, autism spectrum disorders and obsessive–compulsive disorder [2–4]. Full length dystrophin (Dp427) is expressed in the brain, alongside two shorter isoforms of the dystrophin protein, Dp140 and Dp71. These isoforms share significant sequence conservation beyond N-terminal truncations but show temporospatial differences in expression [5].

Animal models of DMD enable detailed studies of dystrophin expression, function and the consequences of its deficiency [6–10]. Dogs represent a valuable model of DMD as the severity and progression of muscle disease closely mimics that of humans [11] and their brain size and structure is more comparable to humans than those of rodents. Dp427, Dp140 and Dp71 dystrophin isoforms are expressed in the normal adult canine brain with regional expression patterns that reflect those of the human brain [12]. In 2010, we reported a spontaneous splice site mutation in a Cavalier King Charles Spaniel that results in deletion of exon 50 and an out of frame transcript [8]. This mutation site falls within the major human DMD mutational hotspot (exons 45–53) and has since been maintained on a beagle background to create the DE50-MD dog model of DMD. DE50-MD dogs are deficient in brain Dp427 protein expression but retain expression of Dp140 and Dp71, albeit with a mild reduction in Dp140; these dogs have a detectable cognitive and neurobehavioral phenotype including reduced attention, problem solving and exploration of novel objects [12].

A range of imaging modalities has been used to evaluate the dystrophin-deficient brain, including magnetic resonance imaging (MRI), magnetic resonance spectroscopy (MRS) and positron emission tomography (PET). In the *mdx* mouse model of DMD (deficient in Dp427), an increase in gray matter volume compared to wildtype (WT) control mice, including the hippocampus and globus pallidus, was identified on MRI performed postmortem [13]. In the *mdx52* mouse model (deficient in Dp427 and Dp140) no gross structural changes were identified in the brain when 12 areas of interest were assessed by MRI and histological analysis [14]. In patients with DMD, significant global morphological and microstructural differences were detected using MRI, specifically reductions in total brain volume, gray matter volume and white matter microstructural integrity as well as a reduction in cerebral perfusion [15–17]. Intracranial volume was smaller in DMD patients compared to healthy controls, but this difference failed to reach statistical significance [16].

The aim of this study was to perform quantitative evaluation of MRI of the brain of DE50-MD dogs and WT age matched controls to compare gray matter, white matter and ventricle volume. Additionally, we used computed tomography (CT) to evaluate skull size and dimensions. We reveal that DE50-MD dogs have a reduction in regional gray matter volumes, with larger lateral ventricles compared to age-matched WT controls.

## Results

**Fig. 1:**
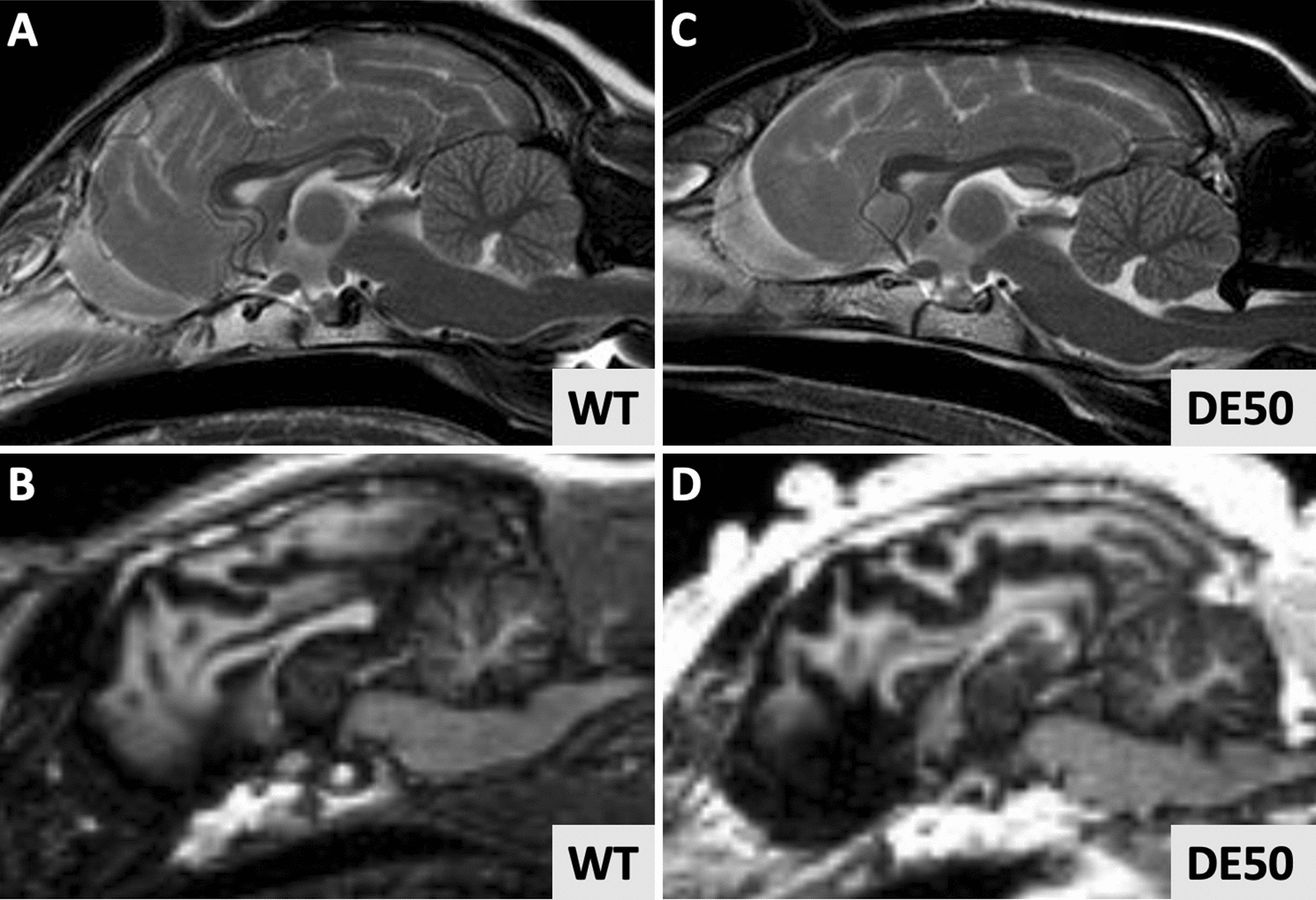
Representative sagittal magnetic resonance images from a 18mo WT dog **A**, **B** and 18mo DE50-MD dog **C**,**D**. (Sagittal plane images, **A**,**C**: T2-weighted, **B**,**D**: T1-weighted). No gross structural differences were noted between DE50-MD and age-matched WT controls

Initial evaluation of MR images did not identify gross structural abnormalities in any of the studied dogs (Fig. 1).

### Principal component analysis (PCA)

**Fig. 2:**
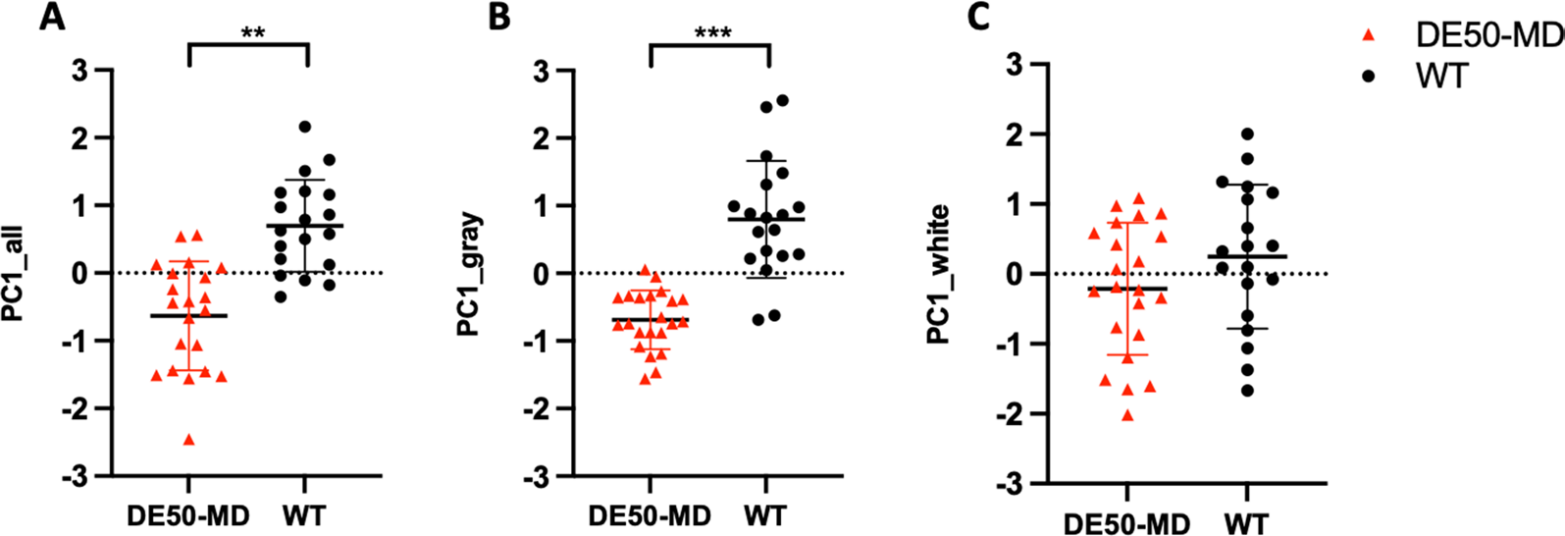
Principal component analysis of MRI variables in DE50-MD and WT dogs. **A** Principal component 1 (PCA1) was significantly different between DE50-MD and WT dogs when all evaluated MRI variables were included. **B** When all gray matter variables were evaluated PCA1 (PCA1_gray) was significantly different between DE50-MD and WT dogs. **C** When all white matter variables were evaluated, there was no difference in PCA1 (PAC1_white) between DE50-MD and WT dogs (*p < 0.05, **p < 0.01, ***p < 0.001). Individual data, mean and standard deviation shown

A PCA was performed to evaluate all MRI variables for all dogs. PCA revealed nine components with an Eigenvalue greater than one, accounting for 21.4%, 13.6%, 11.5% and 8.6%, 7.0%, 5.7%, 5.1%, 4.2% and 3.5% of the total variance, respectively. For Principal component 1 (PC1_all), there was a significant difference between DE50-MD dogs and WT (p = 0.002) (Fig. 2A), but not between ages (p = 0.577) nor their interaction (p = 0.293). Cingulate gyrus volume, piriform lobe volume and lateral ventricle volume contributed most to PC1.

A PCA was then performed with only the gray matter variables, revealing six components with an Eigenvalue of greater than one. PC1_gray accounted for 24.2% of the total variance, with cingulate gyrus, piriform lobe and hippocampus contributing most. Again, there was a significant difference in PC1_gray between DE50-MD dogs and WT (p =  < 0.001) (Fig. 2B), but not between ages (p = 0.515).

Finally, a PCA was performed with only the white matter variables, revealing two components with an Eigenvalue greater than one. PC1_white accounted for 35.6% of the total variance. No significant difference was identified in PC1_white between DE50-MD dogs and WT dogs (p = 0.468) (Fig. 2C).

### Linear mixed model of individual MRI measurements

**Fig. 3:**
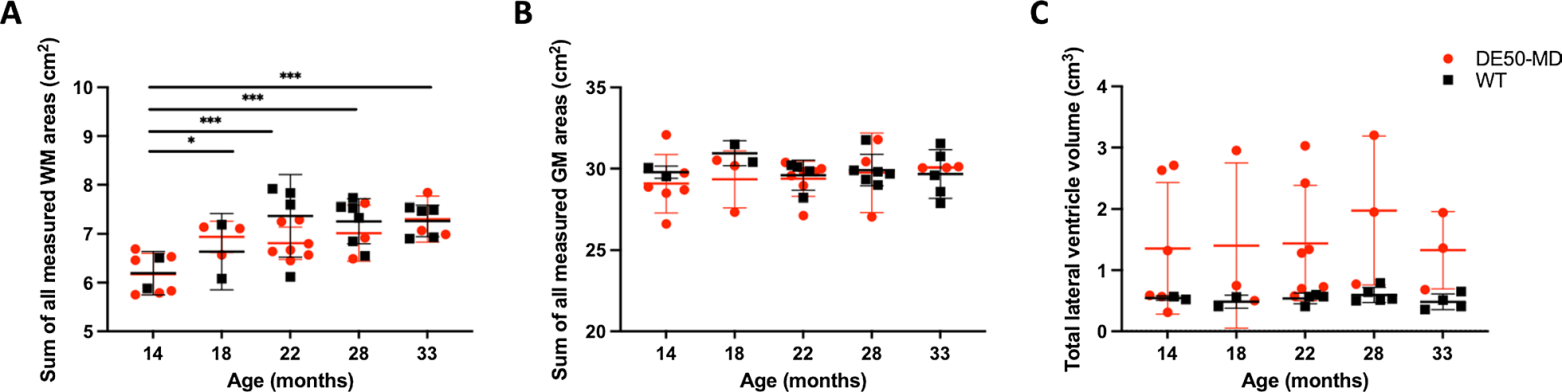
Total recorded white matter area showed a significant increase with age in both DE50-MD and WT dogs (**A**), while total recorded gray matter area (**B**) and total lateral ventricle volume (**C**) remained constant across studied ages (*p < 0.05, ***p < 0.001). Individual data, mean and standard deviation shown

No significant interaction was found when data were grouped by genotype and age (i.e. DE50MD 6 m, WT 6 m) for any of the studied variables. When grouped by age alone, the summative value of all recorded white matter areas was significantly associated with age (p = 0.001), with an increase in white matter area detected in both DE50-MD and WT dogs with age (Fig. 3A). All other measured variables showed no significant interaction with age; summative gray matter area and lateral ventricle volume showed no change over the studied ages (Fig. 3B, C).

**Fig. 4:**
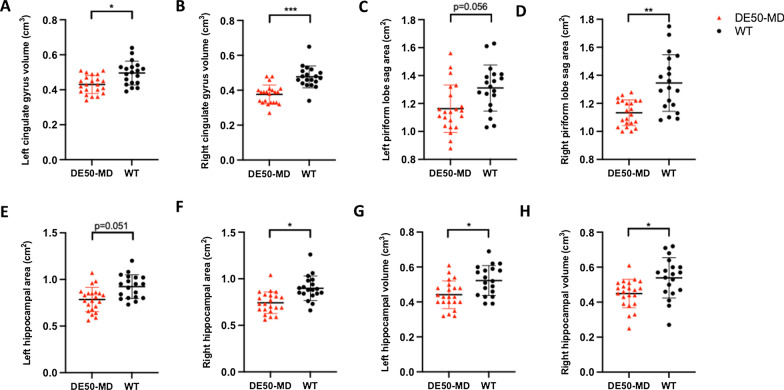
MRI revealed regional reductions in gray matter in DE50-MD dogs compared with WT on T2-weighted images. **A** Left and **B** right cingulate gyrus volume, **C** left and **D** right piriform lobe area, **E** left and **F** right hippocampal area and **G** left and **H** right hippocampal volume were decreased in DE50-MD dogs compared with WT (*p < 0.05, **p < 0.01, ***p < 0.001). Individual data, mean and standard deviation shown

On T2w images, when all data points were grouped by genotype, the cingulate gyrus volumes (left p = 0.015, right p < 0.001), right piriform lobe area (p = 0.007), right hippocampal area (p = 0.043) and hippocampal volumes (left p = 0.01, right p = 0.044) were significantly lower in DE50-MD dogs compared with WT (Fig. 4).

**Fig. 5:**
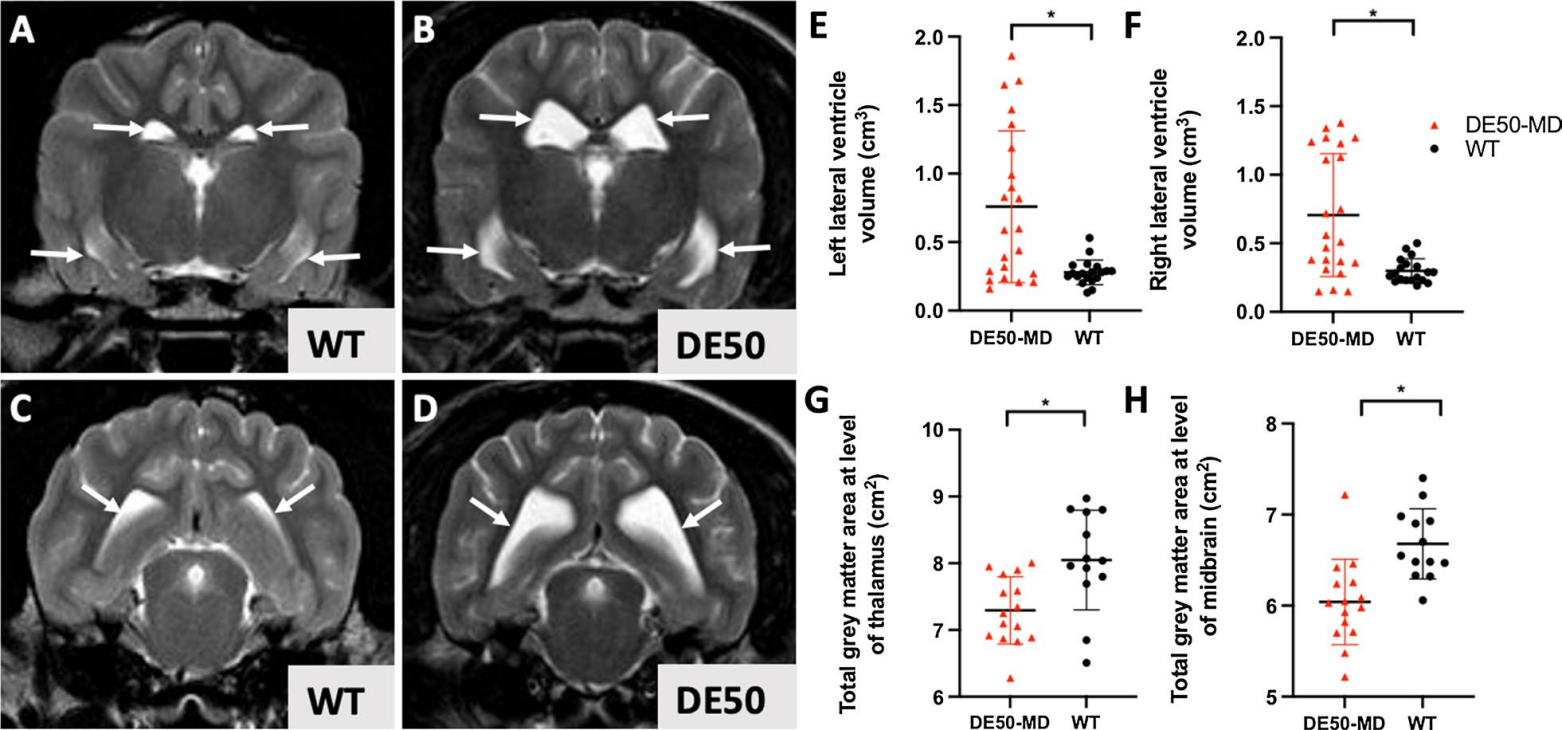
**A**–**F** Lateral ventricle volume was significantly higher in DE50-MD dogs compared with WT. Representative T2-weighted transverse magnetic resonance images of the brain of a 28mo WT dog and 28mo DE50-MD dog shows comparatively larger lateral ventricles (indicated by white arrows) in the DE50-MD dog (**C**,**D**). (**A**,**C**: at the level of the caudal aspect of the third ventricle. **B**, **D**: at the level of the caudal colliculi). Both the left (**E**) and right (**F**) lateral ventricles were larger in volume in DE50-MD dogs compared with WT (*p < 0.05). **G**, **H**) T1W images revealed reduced regional gray matter in DE50-MD dogs compared with WT. **G**) Cerebral cortical gray matter transverse area at the level of the interthalamic adhesion and **H**) at the level of the rostral colliculi of the midbrain was significantly decreased in DE50-MD dogs compared with WT (*p < 0.05). Individual data, mean and standard deviation shown

The lateral ventricle volumes showed higher variation in DE50-MD dogs (range 0.15–1.86 cm^3^, compared to 0.19–0.53 cm^3^ in WT), with a significantly greater volume compared to WT (left p = 0.045, right p = 0.036) (Fig. 5A–F).

On T1w images, cerebral cortical gray matter transverse area at the level of the ITA (p = 0.033) and at the level of the rostral colliculi of the midbrain (p = 0.015) were significantly decreased in DE50-MD dogs (Fig. 5G, H). Other measured variables showed no difference with age nor genotype.

### Linear mixed model of skull CT measurements

Maximum internal skull height, maximum internal skull width and total volume of the intracranial vault as measured on CT was not different between DE50-MD and WT dogs (Additional file 1: Table S1).

## Discussion

Approximately 50% of patients with DMD have intellectual disability and/or neurobehavioural comorbidities due to dystrophin deficiency in the brain [1, 18]. Similarly, altered brain function has been documented in mouse and dog models of DMD [12, 19–23]. In this study we used MRI to study the brain of DE50-MD dogs, a canine model of DMD that is deficient in Dp427 protein expression and has a modest reduction in Dp140 protein expression. This revealed regional gray matter reduction in the brains of dystrophic dogs when compared with age-matched WT dogs. Reduced gray matter was detected in the piriform lobe, hippocampus and cingulate gyrus with enlargement of the lateral ventricles. White matter volume and cranial vault dimensions were unchanged.

Studies of the *mdx* mouse model of DMD, which carries a point mutation in exon 23 and fails to express full length dystrophin protein (Dp427), have shown a non-significant increase in brain volume compared to WT controls [13, 24, 25]. Specifically, the hippocampus, globus pallidus and caudate putamen were larger in *mdx* mice [13]. This contrasts markedly with the reduction in gray matter volume reported in DMD patients [16, 26] and to the reduced regional gray matter volumes we have identified in the DE50-MD canine model of DMD. Therefore, in comparison to mouse models, DE50-MD dogs might offer a more translationally relevant pre-clinical model to study the consequences of brain dystrophin deficiency.

The limbic system consists of a complex group of closely interrelated brain structures that have a broad influence on brain function, including emotion and behaviour. Limbic structures include the hippocampus, cingulate gyrus, parahippocampal gyrus of the piriform lobe, mammillary bodies of the hypothalamus, thalamus, amygdala and septal nuclei. Our findings of a reduction in volume of the hippocampus, cingulate gyrus and piriform lobe might reflect disruption of the limbic system and thus contribute to the identified neurocognitive phenotype [12]. In DMD patients, imaging studies have identified changes consistent with altered limbic system function: functional MRI revealed hyperconnectivity in the default mode network [27] that includes the prefrontal cortex, cingulate cortex and hippocampus, and PET showed glucose hypometabolism of the hippocampus [28]. The *mdx* mouse exhibits exaggerated startle responses to threat [20, 29], linked to the deficiency of dystrophin in limbic structures such as the amygdala. Furthermore, enhanced fearfulness was associated with reduced exploration of novel objects when assessed in an open field test [29]. A reduction in GABA-A receptor clustering and disrupted inhibitory synaptic function has been detected in the amygdala and hippocampus of the *mdx* mouse and is considered central to the enhanced fear responses [30, 31]. Anxiety symptoms are reported by 24–33% of people with DMD [2, 32, 33], particularly increased fear-based anxiety symptoms (social and separation anxiety) [34]. A recent study identified an increased unconditioned startle response in boys with DMD [35]. Furthermore, Dp427 (cortical and muscle isoforms) is highly expressed in the human hippocampus and amygdala [5]. Assessment of anxiety and enhanced fear phenotype in the DE50-MD dogs, along with interrogation of GABA-receptor clustering, will be a key goal for future studies.

Musculoskeletal impairment has been associated with alterations in brain regions responsible for the production and perception of movement in people [36, 37]. Hence, it is possible that the skeletal muscle pathology in DMD results in secondary changes in brain motor centres. In this study we have not specifically interrogated the motor centres given the challenges in accurately delineating these in the canine brain. A goal of future studies will be to evaluate motor skills and compare these to volumetric and functional MRI assessments of cortical and subcortical motor centres in DE50-MD dogs.

We found no significant differences in white matter volume between DE50-MD dogs and WT controls. This was also reported in boys with DMD and healthy matched controls [16]. However, diffusion tensor imaging revealed white matter changes at a microstructural level, with smaller fractional anisotropy and increased medial diffusivity, indicative of compromised structural complexity [17]. Unfortunately, diffusion tensor imaging is not supported with our current MRI facilities and hence we were unable to evaluate white matter integrity but this will be a goal for future studies. We previously reported reduced interaction with a novel olfactory cue, with failure to habituate to a mirror reflection in DE50-MD dogs [12], potentially consistent with compromise of the prefrontal cortex and frontal cortex circuitry. Future studies to interrogate white matter integrity of the prefrontal cortex and frontal circuitry might identify structural correlations to the detected neurocognitive phenotype.

Reduced total brain volume in DMD patients compared with healthy age-matched controls has been reported, with no significant change in cerebrospinal fluid (CSF) volume [16]. Furthermore, skull size was found to be proportional to total brain volume, suggesting that the smaller brains in DMD are a result of differences in maturation rather than atrophy. In dogs, we found no decrease in total brain volume, but an increase in volume of the lateral ventricles, raising the possibility that reduced gray matter volume was compensated for by an increase in CSF volume so maintaining stable total brain volume within the unchanged skull volume. A study of *mdx* mice similarly identified an increase in lateral ventricle volume [25]. Given the consistent gray matter volumes over the studied age range, failure of gray matter development would appear more likely than a progressive atrophy in the DE50-MD dogs. Studies to interrogate the histopathological changes that result in reduced gray matter volume alongside ventricular enlargement might reveal insights into the cause of these changes at the cellular level and so provide further insights on the precise consequences of dystrophin deficiency in the canine brain. Furthermore, the gray matter changes appear to be established by 14 months of age in our model, suggesting that therapeutic interventions aiming to increase Dp427 expression and prevent development of the neurocognitive phenotype would require earlier administration. Characterisation of the precise timing at which the gray matter changes arise would facilitate identification of a potential therapeutic window for future interventional studies.

The DE50-MD dog is deficient in Dp427, but also shows a modest reduction in Dp140 protein expression in the brain [12]. Boys deficient in both Dp427 and Dp140 have been found to have smaller gray matter, total brain and intracranial volumes compared to boys deficient in Dp427 only [16]. The identified regional gray matter reductions in the DE50-MD dog could therefore be a consequence of both the Dp427 deficiency and of the modest reduction in Dp140. Characterisation of which isoforms are expressed in the piriform lobe, hippocampus and cingulate gyrus, and to what extent, could help ascertain the relative roles of these isoforms in the identified gray matter changes.

Corticosteroids are often used in patients with DMD to delay the progression of muscle weakness and prolong ambulation, but their use has been associated with detectable changes on brain MRI. For example, reductions in hippocampal and amygdala volume are documented in patients treated with steroids for rheumatoid arthritis or asthma [38] and children with hyperadrenocorticism (elevated endogenous steroid production) have a significantly reduced total brain and amygdala volume, with larger ventricles compared with controls [39]. DE50-MD dogs are steroid naïve and so the identified reduction in regional gray matter is not a consequence of concurrent corticosteroid treatment.

Limitations of the study include the small sample size, the variability in the number and timing of the imaging studies performed in each dog and the relatively short time window over which imaging studies were performed (14–36 months old). We have not yet been able to develop and perform segmentation analysis in the canine brain to enable quantification of total gray matter volume, total white matter volume and total CSF volume. Analysis of T1w images identified a reduction in gray matter transverse area at two locations in the DE50-MD dog brain (ITA and rostral colliculi), however this was not replicated in T2w images. This could represent differing delineation of the gray and white matter between the two sequences, inaccuracies in assessment and/or inadequate sensitivity or representation of our chosen regions. In boys with DMD, the reduction in gray matter is global [16] and therefore future studies to evaluate large areas of gray matter volume in DE50-MD dogs are needed. Another important limitation of the study is that not all regions of the brain were assessed quantitatively. Regions of particular interest, such as the amygdala and motor cortex cannot be visualised accurately on our MR images and hence volumetric assessment was not possible. The amygdala underlies the rostral portion of the piriform lobe and our findings of a smaller piriform lobe in the DE50-MD dogs might encompass a reduction in size of the amygdala. Segmentation analysis, higher resolution MRI to enable accurate volumetric analysis of further regions of interest, evaluation of white matter integrity with diffusion tensor imaging, evaluation of metabolic status of the brain with MRS and functional MRI studies will provide valuable further comparative insights to the microanatomy and physiology of the Dp427-deficient canine brain.

## Conclusions

The DE50-MD dog model of DMD has regional reductions in gray matter volume with enlargement of the lateral ventricles. These changes are presumably a consequence of absent dystrophin expression in the brain and provide important insights into key brain regions to evaluate for relevant neurobehavioural comorbidities contributing to brain dysfunction in this canine model of DMD.

## Methods

### Animals and anaesthesia

This study was conducted within a UK Animals (Scientific Procedures) Act 1986 (ASPA) project licence with approval by the local Animal Welfare Ethical Review Board (AWERB) and is reported in accordance with ARRIVE guidelines. No dogs were specifically bred for this study; the dogs were bred for a separate natural phenotype study. Carrier female Beagle (RCC strain)-cross dogs (F3 generation) were derived from an original founder female carrier (Bichon-Frise cross Cavalier King Charles Spaniel) purchased by the institution from the breeder to establish a research colony of DE50-MD dogs. Carrier females were mated in house with purchased Beagle stud males (RCC strain, Envigo) to produce WT, carrier and DE50-MD offspring. Dogs were housed in groups of 3–4 (12 h light/dark cycle; 15–24 °C), in a dedicated canine facility with large pens (minimum of 2 × 4.5 m), access to outdoor runs, grass paddocks and different types of enrichment items, conditions that exceed the minimum stipulated by the UK Animal (Scientific Procedures) Act 1987. All animals followed a comprehensive socialisation programme with daily human interactions and were acclimatised to routine procedures.

MRI was performed in 8 DE50-MD dogs and 6 age-matched littermate WT male dogs every 3 to 6 months, from 14 to 33 months of age under general anaesthesia, though not all dogs were included at every time point. CT of the skull was performed in 8 DE50-MD dogs and 5 age-matched littermate WT male dogs every 3 to 6 months, from 18 to 36 months of age under general anaesthesia, but again not all dogs were included at every time point. The sample was limited to dogs available through a separate natural phenotype study; no dogs were bred specifically for this study and all work was conducted in alignment with other studies in accordance with the institute’s policy to minimise experimental dog numbers. Dogs were premedicated with 0.2 mg/kg methadone (Synthadon, Animalcare), induced with 4–6 mg/kg propofol to effect (Propoflo, Zoetis), intubated and maintained on an inhalational mixture of sevoflurane (SevoFlo, Zoetis) and oxygen whilst being infused with 5 ml/kg/hr Hartmann’s solution (Aquapharm11, Animalcare).

### MRI acquisition

Dogs were scanned in dorsal recumbency using a 1.5 T Philips Intera MRI scanner (Philips Medical Systems, Best, The Netherlands). Sagittal and transverse T2-weighted (T2w) TSE sequences were acquired of the head (TE: 120 ms, TR: 3000 ms, slice thickness: 3 mm with 0.3 mm interslice gap). A T1-weighted (T1w) 3D turbo gradient echo sequence was then acquired (TE: 8 ms, TR: 400 ms, slice thickness 0.7–1 mm with 0 mm interslice gap). MR images were analysed by a single investigator (AC) using OsiriX/Horos DICOM viewing software (Free open-source code software, horosproject.org).

The following parameters were measured on T2w images: total intracranial volume, maximal brain sagittal height, length and area, cingulate gyrus volume, cortical gray and white matter transverse areas (at level of interthalamic adhesion (ITA) and at rostral colliculi), thalamus transverse area (at ITA), piriform lobe area (parasagittal), hippocampal transverse area (at rostral colliculi), hippocampal volume, midbrain transverse area (at rostral colliculi), cerebellar gray and white matter volumes, cerebellar sagittal area, brainstem sagittal area, lateral ventricle volume and mesencephalic aqueduct transverse area (at rostral colliculi). The following parameters were measured on T1w images: maximal brain sagittal height, length and area, caudate nuclei volume, thalamic transverse area (at ITA), cortical white and gray matter transverse areas (at ITA and at rostral colliculi), midbrain transverse area (at rostral colliculi), cerebellar gray and white matter volumes and cerebellar sagittal area. These specific regions were selected based on ease of delineation and interest based on previous published literature. Outlines of each area of interest were manually delineated. To calculate volumes, area of interest was manually delineated on each slice and a volume interpolation method used. The mean of 3 repeat measurements was used for statistical analysis.

### CT acquisition

Immediately following MRI, dogs underwent CT in sternal recumbency using a Canon Aquilion ONETM/GENESIS Edition 320-slice CT scanner (Canon Medical Systems, Otawara, Japan). CT images were analysed using a bone window (level: 400HU, width: 1800HU, slice thickness: 0.5 mm) in OsiriX/Horos DICOM viewing software with reconstructions in sagittal and dorsal planes. The following parameters were measured: maximum internal skull width and height, and total intracranial volume (volume assessment was performed at the 36 months only). To calculate volumes, an outline of the intracranial vault was manually delineated in every 5th slice and regions of interest were generated for the intervening slices; these were manually altered to the correct area on each slice as needed. The mean of 5 repeat measurements was used for statistical analysis.

### Statistical analysis

Data were assessed for normality using the Shapiro Wilk Test and assessment of frequency histograms. A principal component analysis (PCA) was used to evaluate overall variation in the recorded MRI parameters among the DE50-MD and WT groups and across all ages. The effects of age, genotype and their interaction on the first component (PC1) was examined statistically using a linear mixed model (LMM) with Fisher’s LSD post-hoc comparisons. To account for repeated measures in the LMM analysis, dog was included as a random effect. The effects of age, genotype and their interaction on each recorded MRI and CT variable were then examined statistically, again using a LMM with Fisher’s LSD post-hoc comparisons with adjustment for multiple comparisons. Statistical analysis was performed using SPSS software (IBM SPSS Statistics 28) and results are expressed as mean ± SD unless otherwise stated. Differences and associations were considered statistically significant when p < 0.05.

## Supplementary Information

**Additional file 1: Table S1. **Computed tomography measurements of skull height, width and cranial vault volume.

## Data Availability

The data that support the findings of this study are available from the corresponding author on reasonable request.

## Abbreviations

CT: Computed tomography
CSF: Cerebrospinal fluid
DMD: Duchenne muscular dystrophy
DE50-MD: Delta50 canine model of muscular dystrophy
HU: Hounsfield units
ITA: Interthalamic adhesion
LMM: Linear mixed model
MRI: Magnetic resonance imaging
MRS: Magnetic resonance spectroscopy
PCA: Principal component analysis
PC1: Principal component 1
PET: Positron emission tomography
SD: Standard deviation
WT: Wildtype
