# Supplementary material for: Brain magnetic resonance imaging in the DE50-MD dog model of Duchenne muscular dystrophy reveals regional reductions in cerebral gray matter
Source: BMC Neurosci. 2023 Mar 17;24:21. doi: 10.1186/s12868-023-00788-2 (PMC10024360; doi:10.1186/s12868-023-00788-2)
Supplement: Supplementary file 1 — Additional file 1: Table S1. Computed tomography measurements of skull height, width and cranial vault volume. [file 12868_2023_788_MOESM1_ESM.docx]

**Additional Information**

**Additional Table 1:** Computed tomography measurements of skull height, width and cranial vault volume.

|  | DE50-MD | WT | P value |
| --- | --- | --- | --- |
| Internal skull height (cm) | 4.25±0.14 | 4.37±0.22 | 0.775 |
| Internal skull width (cm) | 4.96±0.17 | 4.95±0.14 | 0.772 |
| Intracranial vault volume (cm^3^) | 79.64±1.17 | 75.44±3.76 | 0.078 |

**Additional Table 1:** CT measurements of the height, width and cranial vault volume of the skull revealed no differences between DE50-MD and WT dogs. Data shown as mean and standard deviation (n=8 DE50-MD, n=5 WT). CT: computed tomography, DE50-MD: canine model of Duchenne muscular dystrophy, WT: wildtype.
